# Rapamycin inhibits pathogen transmission in mosquitoes by promoting immune activation

**DOI:** 10.1371/journal.ppat.1009353

**Published:** 2021-02-24

**Authors:** Yuebiao Feng, Lu Chen, Li Gao, Li Dong, Han Wen, Xiumei Song, Fang Luo, Gong Cheng, Jingwen Wang

**Affiliations:** 1 The State Key Laboratory of Genetic Engineering, School of Life Sciences, Fudan University, Shanghai, China; 2 Ministry of Education Key Laboratory of Contemporary Anthropology, School of Life Sciences, Fudan University, Shanghai, China; 3 Tsinghua-Peking Joint Center for Life Sciences, Beijing Advanced Innovation Center for Structural Biology, School of Medicine, Tsinghua University, Beijing, China; University of Georgia, UNITED STATES

## Abstract

Repeated blood meals provide essential nutrients for mosquito egg development and routes for pathogen transmission. The target of rapamycin, the TOR pathway, is essential for vitellogenesis. However, its influence on pathogen transmission remains to be elucidated. Here, we show that rapamycin, an inhibitor of the TOR pathway, effectively suppresses *Plasmodium berghei* infection in *Anopheles stephensi*. *An*. *stephensi* injected with rapamycin or feeding on rapamycin-treated mice showed increased resistance to *P*. *berghei* infection. Exposing *An*. *stephensi* to a rapamycin-coated surface not only decreased the numbers of both oocysts and sporozoites but also impaired mosquito survival and fecundity. Transcriptome analysis revealed that the inhibitory effect of rapamycin on parasite infection was through the enhanced activation of immune responses, especially the NF-κB transcription factor REL2, a regulator of the immune pathway and complement system. Knockdown of *REL2* in rapamycin-treated mosquitoes abrogated the induction of the complement-like proteins *TEP1* and *SPCLIP1* and abolished rapamycin-mediated refractoriness to *Plasmodium* infection. Together, these findings demonstrate a key role of the TOR pathway in regulating mosquito immune responses, thereby influencing vector competence.

## Author summary

Anautogenous mosquitoes must consume vertebrate blood meals to complete oogenesis. Repeated blood feeding makes the mosquitoes efficient disease-transmitting vectors. The TOR pathway activated by ingested blood is known as an important regulator for vitellogenesis in mosquitoes. Herein, we show that the protein kinase TOR is involved in the regulation of mosquitoes’ susceptibility to *Plasmodium* infection. Inhibition of the TOR pathway by rapamycin upregulates the expression of REL2, a transcription factor controlling the expression of a variety of immune effectors. The enhanced immune responses in turn promote parasite elimination. Therefore, the TOR pathway plays a dual role in not only regulating mosquito reproduction but also in their vector potential.

## Introduction

Repeated blood meals provide nutrients for egg development and also make mosquitoes efficient disease-transmitting vectors [1]. *Plasmodium* sp. transmitted by *Anopheles* mosquitoes caused 435,000 deaths globally [2]. Ingestion of *Plasmodium* induces profound changes in mosquitoes, with approximately 3%-8% of the total mosquito transcriptome being differentially regulated [3, 4]. These genes are involved in multiple physical processes, including apoptosis, immunity, metabolism, cell structure, and cell adhesion. The coordination of changes in development, metabolism, and immunity reveals complex host-pathogen interactions. However, how the mosquito adjusts the metabolic and immune system in response to pathogen infection remains to be determined.

The target of rapamycin, the TOR signaling pathway, is conserved from yeast to mammals and integrates extracellular and intracellular nutrients and growth factors to regulate cell metabolism, growth, and proliferation [5]. Mosquito TOR signaling is a key pathway that controls vitellogenesis in response to blood feeding [6]. Proteins are the predominant constituents of blood. Approximately 12% of the blood meal-derived amino acids are used for vitellogenin synthesis [7]. Within hours after a blood meal, there is a significant increase in hemolymph amino acid levels [8]. This increase leads to the activation of the TOR signaling pathway. TOR activation phosphorylates S6 kinase (S6K) and the translational repressor 4E-Binding Protein (4E-BP), ultimately stimulating protein translation and initiating egg development [6, 9–13]. Rapamycin, an inhibitor of the TOR pathway, effectively suppresses vitellogenesis in mosquitoes [6]. As a central node that integrates different metabolic cues from the microenvironment, the role of the TOR pathway in pathogen transmission in mosquitoes remains to be determined.

In this study, we show that suppression of the TOR pathway in *An*. *stephensi* by rapamycin effectively inhibits *P*. *berghei* infection. Rapamycin treatment induces the expression of the transcription factor *REL2*. The enhanced expression of *REL2* upregulates the expression of multiple immune effectors, including *TEP1* and *SPCLIP1*, which promotes parasite elimination.

## Results

### Inhibition of the TOR pathway promotes the defense of *An*. *stephensi* against *P*. *berghei*

**Fig 1:**
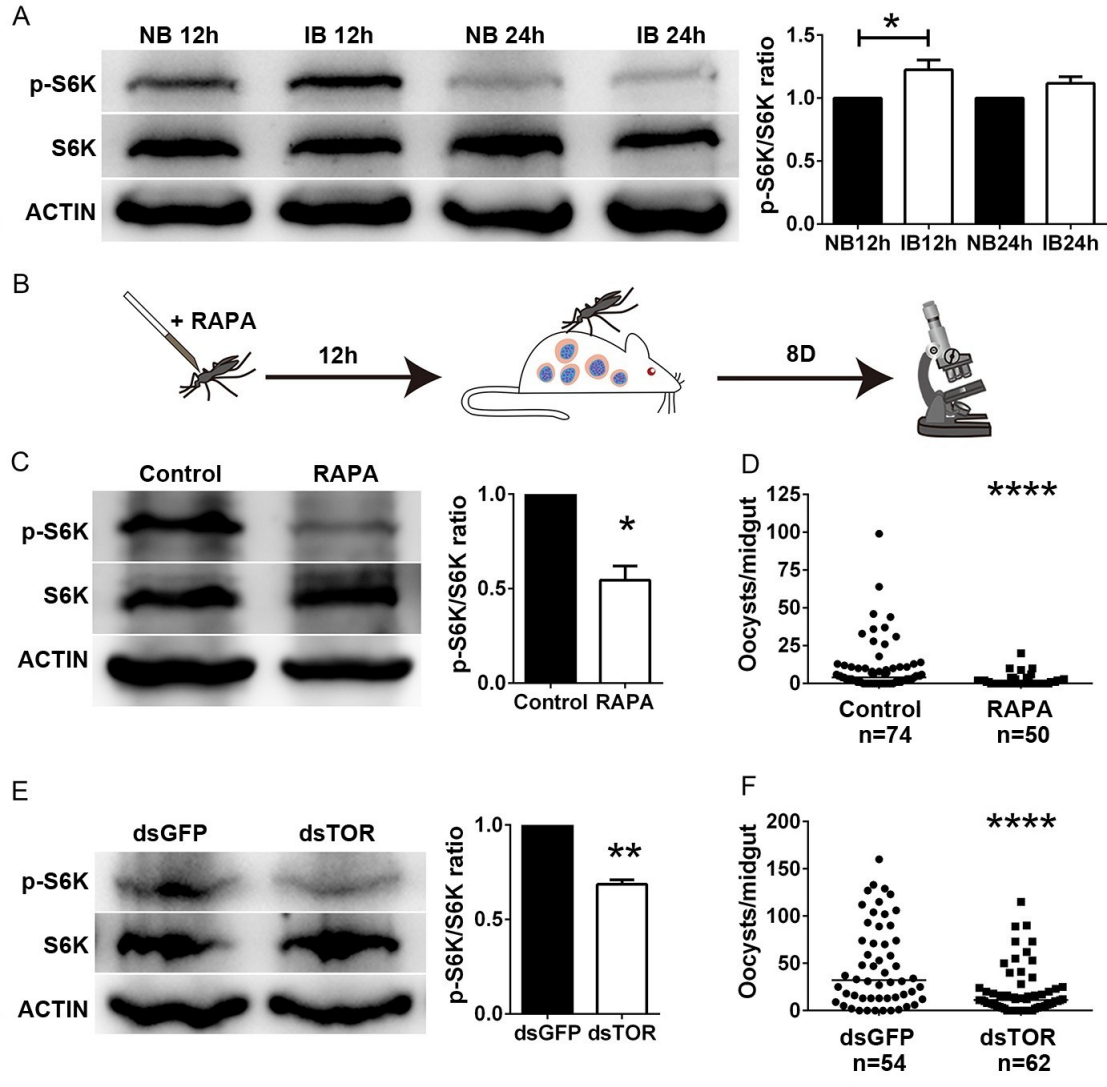
Inhibition of the TOR pathway decreases *P*. *berghei* infection. (A) Influence of *P*. *berghei* infection on the TOR pathway by Western blot analysis. Phosphorylation levels of S6K from fat bodies 12 h and 24 h post-normal (NB) and infectious blood meal (IB) were analyzed using anti-Phospho-S6K antibody. *An*. *stephensi* S6K and ACTIN were used as internal controls (left panel). The intensity of phosphorylated S6K was normalized to that of S6K. The relative intensity of phosphorylated S6K in IB-fed mosquitoes was normalized to that of NB-fed mosquitoes (right panel). Results were pooled from three independent experiments. (B) Schematic overview of rapamycin microinjection of *An*. *stephensi*. Vehicle solution was injected as a control. (C) The influence of rapamycin treatment on the mosquito TOR pathway. Phosphorylation levels of S6K from fat bodies 24 h post-blood meal were analyzed. Quantification of p-S6K signal intensity in rapamycin-treated mosquitoes was normalized to that of controls. The results are from two independent experiments. (D) Influence of rapamycin treatment on *Plasmodium* infection. Data were pooled from three independent replicates. (E) Influence of *TOR* knockdown on the activity of the TOR signaling pathway. Relative quantification of p-S6K signal intensity was from two independent replicates. (F) Influence of *TOR* knockdown on *Plasmodium* infection. The data were pooled from three independent biological experiments. Horizontal black bars indicate median oocyst numbers. Each dot represents an individual mosquito. Error bars indicate standard errors. Significance was determined by Student’s *t-*test in (A), (C), and (E) and by Mann-Whitney tests in (D) and (F); *P<0.05, **P<0.01, ****P<0.0001.

The mosquito TOR pathway is responsible for initiating egg development [6]. *Plasmodium* infection reduces fecundity in multiple mosquito species [14, 15]. To examine whether the compromised fecundity in *Plasmodium* infected mosquitoes could be caused by the dysregulation of the TOR pathway, we analyzed the TOR activity by Western blot analysis. The phosphorylation level of ribosomal S6 kinase (S6K), a TOR substrate, was used as the indicator of TOR activity [16]. Fat bodies were collected at 12 h and 24 h post-feeding from mosquitoes that fed on mice infected with *P*. *berghei* or on uninfected mice. Infectious blood meals increased the phosphorylation level of S6K in mosquitoes at 12 h post-infection (hpi) compared to those that fed on normal blood (Fig 1A). We next examined whether the TOR pathway could influence parasite infection in *An*. *stephensi*. Mosquitoes were injected intra-thoracically with rapamycin, then allowed to feed on *P*. *berghei* infected mice 12 h post-injection (Fig 1B). Rapamycin treatment strongly decreased the phosphorylation levels of S6K at 24 hpi (Fig 1C). Suppression of TOR resulted in a significant decrease in the number of oocysts compared to the vehicle solution-treated controls (Fig 1D). Given that *Plasmodium* lacks the *TOR* ortholog and that rapamycin treatment has no effect on *Plasmodium* development [17, 18], it is highly possible that rapamycin limits *Plasmodium* infection via inhibition of the mosquito TOR signaling pathway. To address this specifically, we knocked down *TOR* mRNA using a double-stranded (ds) RNA-mediated RNA interference (RNAi) strategy. The dsRNA treatment (dsTOR) led to a 44.5% reduction of *TOR* gene expression and a significant decrease in the protein level of phosphorylated S6K compared to that of the dsGFP controls (Figs S1 and 1E). Similarly, depletion of TOR by RNAi resulted in a significant reduction of the oocyst number, from 32 in dsGFP to 11 in dsTOR (Fig 1F). Altogether, these data suggest that inhibition of the TOR signaling pathway protects *An*. *stephensi* from *Plasmodium* infection.

### Rapamycin treatment in mice prevents *P*. *berghei* infection in *An*. *stephensi*

**Fig 2:**
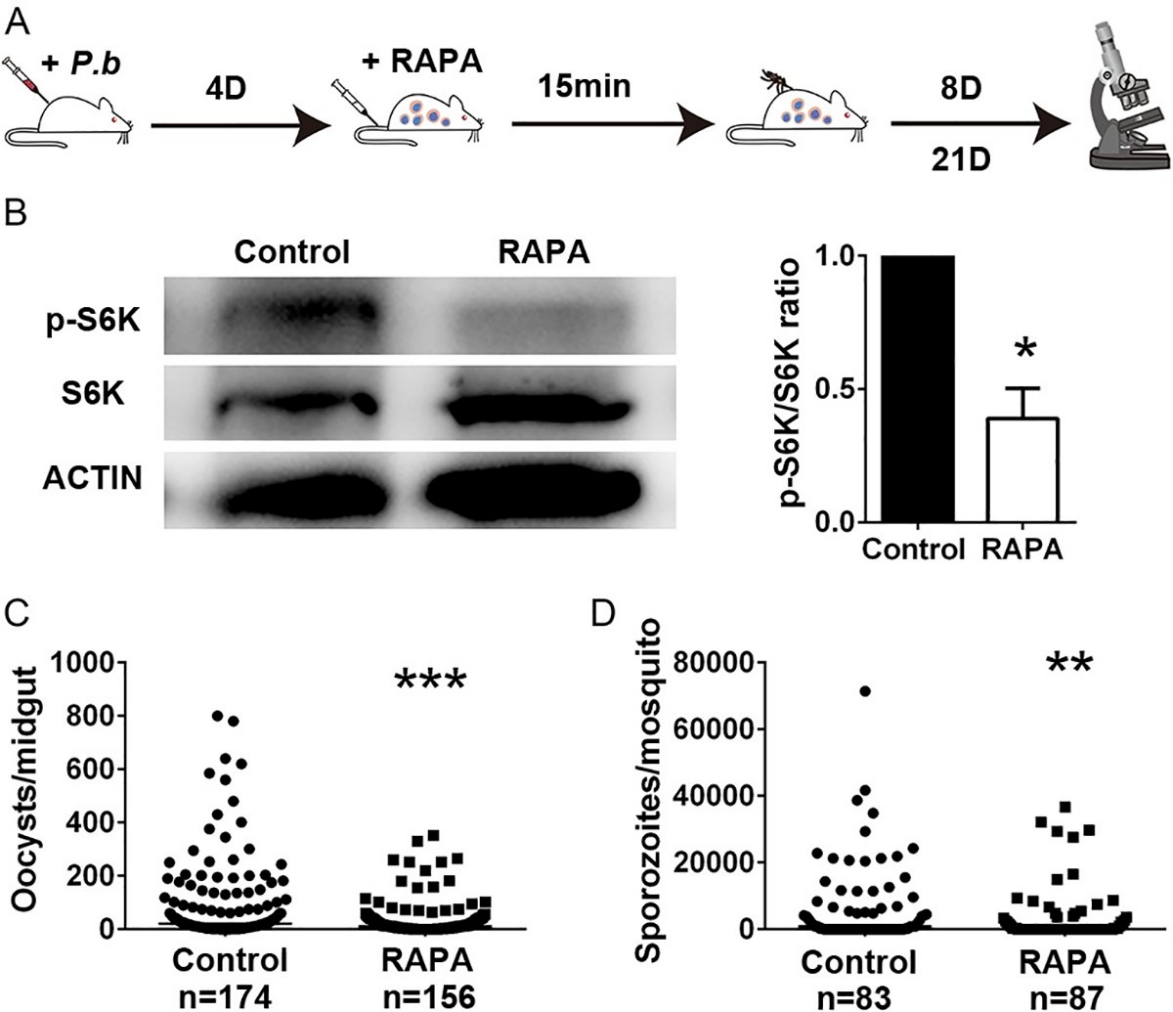
Feeding on rapamycin-treated mice influences *P*. *berghei* infection in *An*. *stephensi*. (A) Schematic overview of rapamycin treatment in mice. Vehicle solution-treated mice were used as controls. (B) Western blot analysis of S6K phosphorylation in fat bodies collected from mosquitoes that fed on rapamycin-treated mice. The bar chart represents the relative quantification of signal intensities from two independent replicates as determined by ImageJ software. Error bars indicate standard errors. (C) Oocyst numbers in mosquitoes feeding on rapamycin (RAPA)-injected and control mice. Data were pooled from three independent biological experiments. (D) Sporozoite numbers from mosquitoes feeding on rapamycin (RAPA)-injected and control mice. Data were pooled from two independent biological experiments. Horizontal black bars indicate the median values. Significance was determined by Student’s *t*-test in (B), and by Mann-Whitney tests in (C) and (D); *P<0.05, **P<0.01, ***P<0.001.

Rapamycin is effective in protecting mice against experimental cerebral malaria (ECM) [19, 20]. In combination with our results, which showed that rapamycin inhibits *P*. *berghei* infection in *An*. *stephensi*, this finding spurred us to ask whether rapamycin treatment in mice would influence parasite infection in mosquitoes. Rapamycin (1.0 mg/kg of body weight) was injected intravenously into the tail veins of mice four days post-*Plasmodium* infection to examine its influence on parasite development. Vehicle solution-treated mice were used as controls. Mosquitoes were allowed to feed on these mice 15 min after rapamycin injection. Oocysts and sporozoites were counted 8 days and 21 days post-infection, respectively (Fig 2A). As expected, short-term rapamycin treatment did not influence parasitemia in mice (S2 Fig). Again, *An*. *stephensi* that fed on rapamycin-treated mice had remarkably reduced levels of phosphorylated S6K at 24 hpi (Fig 2B). These mosquitoes had significantly lower numbers of oocysts and sporozoites than mosquitoes fed on control mice (Fig 2C and 2D). These results suggest that rapamycin treatment in *Plasmodium*-infected mice effectively suppresses the TOR pathway and inhibits *Plasmodium* transmission in mosquitoes.

### Exposing *An*. *stephensi* to a rapamycin-coated surface inhibits *P*. *berghei* infection

**Fig 3:**
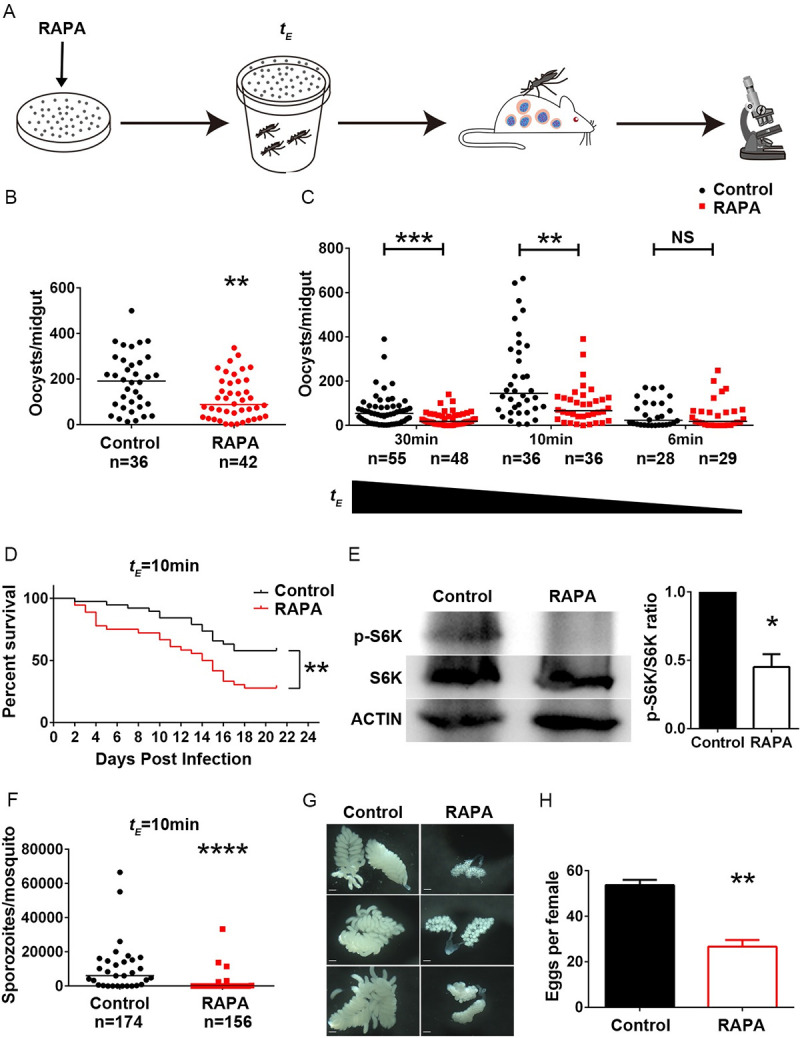
Exposing *An*. *stephensi* to a rapamycin-coated surface inhibits *P*. *berghei* infection, impairs survival and fecundity. (A) Schematic overview of exposing mosquitoes to a rapamycin-treated surface. A solvent-coated surface was used as a control. *t*_*E*_, exposure time. (B) Oocyst numbers in mosquitoes exposed to rapamycin (0.77 mmol/m^2^) (red dots) or solvent (black dots) coated surfaces for 60 min. (C) Mosquitoes were exposed to a 0.77 mmol/m^2^ rapamycin-coated Petri dish for 30 min, 10 min, and 6 min. Data were pooled from two independent replicates (B, C). (D*-*H) Mosquitoes were incubated with a 0.77 mmol/m^2^ rapamycin- or solvent-coated surface for 10 min. (D) Survival curve of mosquitoes exposed to rapamycin (n = 38) or solvent (n = 36) coated surfaces. (E) Western blot analysis of S6K phosphorylation in fat bodies collected from rapamycin-exposed mosquitoes and controls. The bar chart represents relative quantification of signal intensity of p-S6K from two independent replicates as determined by ImageJ software. Error bars indicate standard errors. (F) Sporozoite numbers of rapamycin-treated mosquitoes (red dots) and controls (black dots). Data were pooled from two independent replicates. (G) Ovaries were dissected at 48 h post-normal blood meal from rapamycin-exposed and control mosquitoes. Scale bar, 200 μm. (H) Mean number of eggs laid by 25–35 gravid rapamycin-exposed and control mosquitoes. Data were pooled from three independent replicates. Results from one of three independent experiments are shown (D, G). Horizontal black bars indicate the median values. Significance was determined by Mann-Whitney tests in (B), (C), and (F), by a Log-rank (Mantel-Cox) test in (D), and by Student’s *t*-test in (E) and (H); *P<0.05, **P<0.01, ***P<0.001, ****P<0.0001.

As rapamycin is a lipophilic antibiotic, we speculated that it could be absorbed into the mosquito through penetrating its cuticle and thereby suppress parasite infection. To test this hypothesis, we first exposed *An*. *stephensi* to Petri dishes coated with different concentrations of rapamycin 60 min before infection (Fig 3A). Surprisingly, exposing mosquitoes to rapamycin at 3.85 mmol/m^2^ and 0.77mmol/m^2^ led to a rapid increase of mortality in fully engorged *An*. *stephensi* in comparison to the controls. The surviving mosquitoes displayed increased resistance to *P*. *berghei* infection compared to controls (Figs 3B and S3A). A low concentration of rapamycin (0.077 mmol/m^2^) had no effect on parasite infection (S3B Fig). We next examined the inhibitory effect of 0.77 mmol/m^2^ rapamycin by reducing the exposure time. Mosquitoes incubated with rapamycin for only 10 minutes had significantly lower oocyst numbers than controls (Fig 3C). Again, significantly higher mortality was observed in these mosquitoes than in controls, especially on the first four days post-infection (Fig 3D). Such treatment efficiently blocked TOR activity (Fig 3E). The numbers of sporozoites in the survivors were significantly lower than those in controls (Fig 3F). Inhibiting the TOR pathway via knockdown of *TOR* and *S6K* interrupts egg development in mosquitoes [9, 12]. We next assessed whether rapamycin absorbed by mosquitoes through cuticle penetration could influence *An*. *stephensi* egg development [9, 12]. As expected, exposing mosquitoes to 0.77 mmol/m^2^ rapamycin for 10 minutes before the blood meal strongly inhibited egg development (Fig 3G and 3H). Since it is highly possible that mosquitoes ingested rapamycin during contact with the rapamycin-coated surface, we next examined whether oral administration of rapamycin-containing water would influence *Plasmodium* infection. The oocyst numbers were comparable between rapamycin supplemented and control mosquitoes (S4 Fig). These results suggest that the rapamycin absorbed through cuticle penetration plays a major role in *P*. *berghei* elimination. Taken together, our results indicate that contacting a rapamycin-coated surface effectively inhibits *P*. *berghei* transmission, reduces survival, and impairs egg development in *An*. *stephensi*.

### Inhibition of the TOR pathway by rapamycin changes the immune transcriptional profile in response to *P*. *berghei* infection

**Fig 4:**
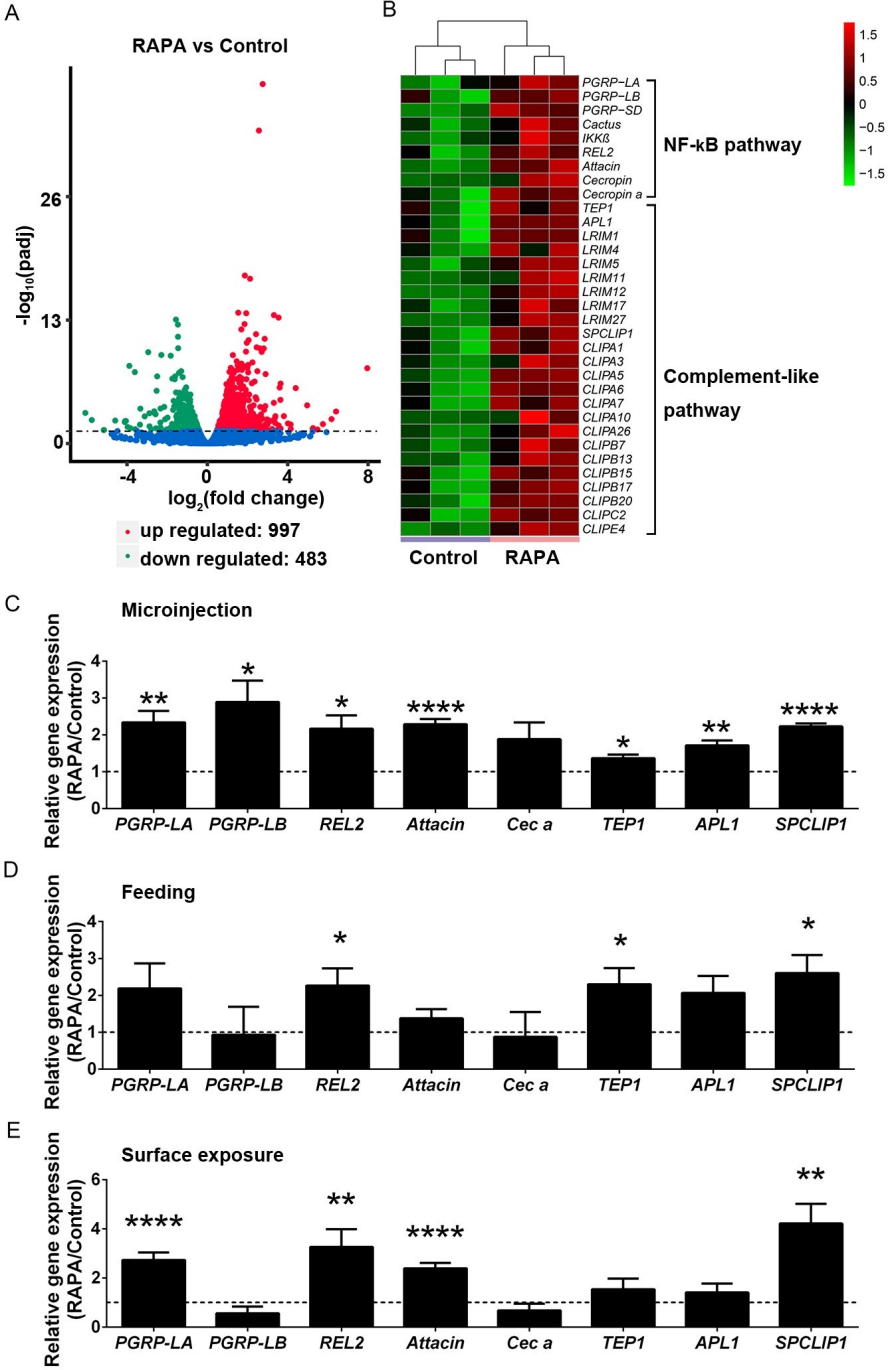
Transcriptome analysis of rapamycin-treated *An*. *stephensi* in response to *Plasmodium* infection. (A) The volcano plot of differentially expressed genes in rapamycin-treated mosquitoes versus controls 24 hpi. Red circles represent 997 significantly up-regulated genes, and green circles represent 483 significantly down-regulated genes (P_adj_ < 0.05). (B) Heat map of immune genes induced by rapamycin. The entire list of differentially expressed genes is shown in S1 Table. (C*-*E) Quantification of upregulated genes by microinjection, feeding, and surface exposure to rapamycin. The expression levels of targeted genes were normalized to *S7*. Relative gene expression in rapamycin-treated mosquitoes was normalized to that of controls. Error bars indicate standard errors (n = 8). Results from one of two independent experiments are shown. Significance was determined by Student’s *t*-test; *P<0.05, **P<0.01, ****P< 0.0001.

To obtain a global view of how suppression of the TOR pathway inhibited *P*. *berghei* infection, we carried out transcriptome analysis of mosquitoes injected with or without rapamycin. The midguts were removed from *Anopheles* 24 hpi. The remaining carcasses were used for RNA-Seq analysis. As expected, rapamycin treatment resulted in a profound transcriptomic change, with 1480 genes differentially regulated (Fig 4A and S1 Table). Gene ontogeny analysis of rapamycin-treated mosquitoes revealed significant enrichment of genes associated with nutrient catabolism processes, including proteolysis, nutrient transport, hydrolase activity, and peptidase activity (S5 Fig). The downregulated expression of several nutrient transporters that are known to facilitate parasite infection, including *Apolipoprotein II/I*, *III*, and *Vitellogenin*, was further verified by qPCR (S6A Fig) [21, 22]. Insulin and TOR signaling pathways regulate metabolism and reproduction synergistically in mosquitoes [13, 23]. Insulin pathway promotes *Plasmodium* infection [24, 25]. We next assessed whether insulin signaling could be involved in the rapamycin mediated increased resistance to *P*. *berghei* infection. The activity of insulin pathway was examined by comparing the phosphorylation levels of serine/threonine kinase AKT (p-AKT) between rapamycin treated and control mosquitoes. No phosphorylated AKT was detected in the midgut, however, rapamycin treatment dramatically reduced the phosphorylation level of AKT in the fat body/ovaries (S6B and S6C Fig). Therefore, the interactions between TOR signaling and other metabolic pathways might also play roles in influencing parasite infection.

In addition, we found a group of immune-related genes, specifically genes associated with NF-κB signaling pathways such as the peptidoglycan recognition proteins (*PGRP-LA*, and *-LB*), NF-κB transcription factor (*REL2*), I-Kappa Kinase (*IKKβ*), antimicrobial peptides (*Attacin* and *Cecropins*), the complement system of leucine-rich repeat proteins (*LRIM1* and *APL1*), thioester-containing protein 1 *(TEP1)*, and CLIP domain serine proteases (*SPCLIP1*), that were significantly induced in rapamycin-treated mosquitoes (Fig 4B). The upregulated immune genes, including *PGRP-LA*, *-LB*, *REL2*, *Attacin*, *Cecropin*, *TEP1*, *APL1*, *and SPCLIP1*, were further validated by quantitative PCR (qPCR) in mosquitoes treated with rapamycin using three different approaches (microinjection, feeding through mice, and surface contact) (Fig 4C–4E). As expected, a similar expression pattern was observed for most of these genes, with *REL2* and *SPCLIP1* significantly induced in all three treatments (Fig 4C–4E). Microbiota is a key factor that determines the immune activity of mosquitoes [26]. It is possible that rapamycin treatment boosts the immune response by influencing the mosquito microbiota. We next quantified the abundance of microbiota in rapamycin-injected mosquitoes. However, no difference in bacterial abundance was observed between rapamycin-treated and control groups (S7 Fig). These results indicate that in addition to influencing mosquito metabolism, the TOR pathway also regulates the immune responses that are responsible for parasite elimination.

### A transcription factor, REL2, is essential in rapamycin-mediated *P*. *berghei* clearance

**Fig 5:**
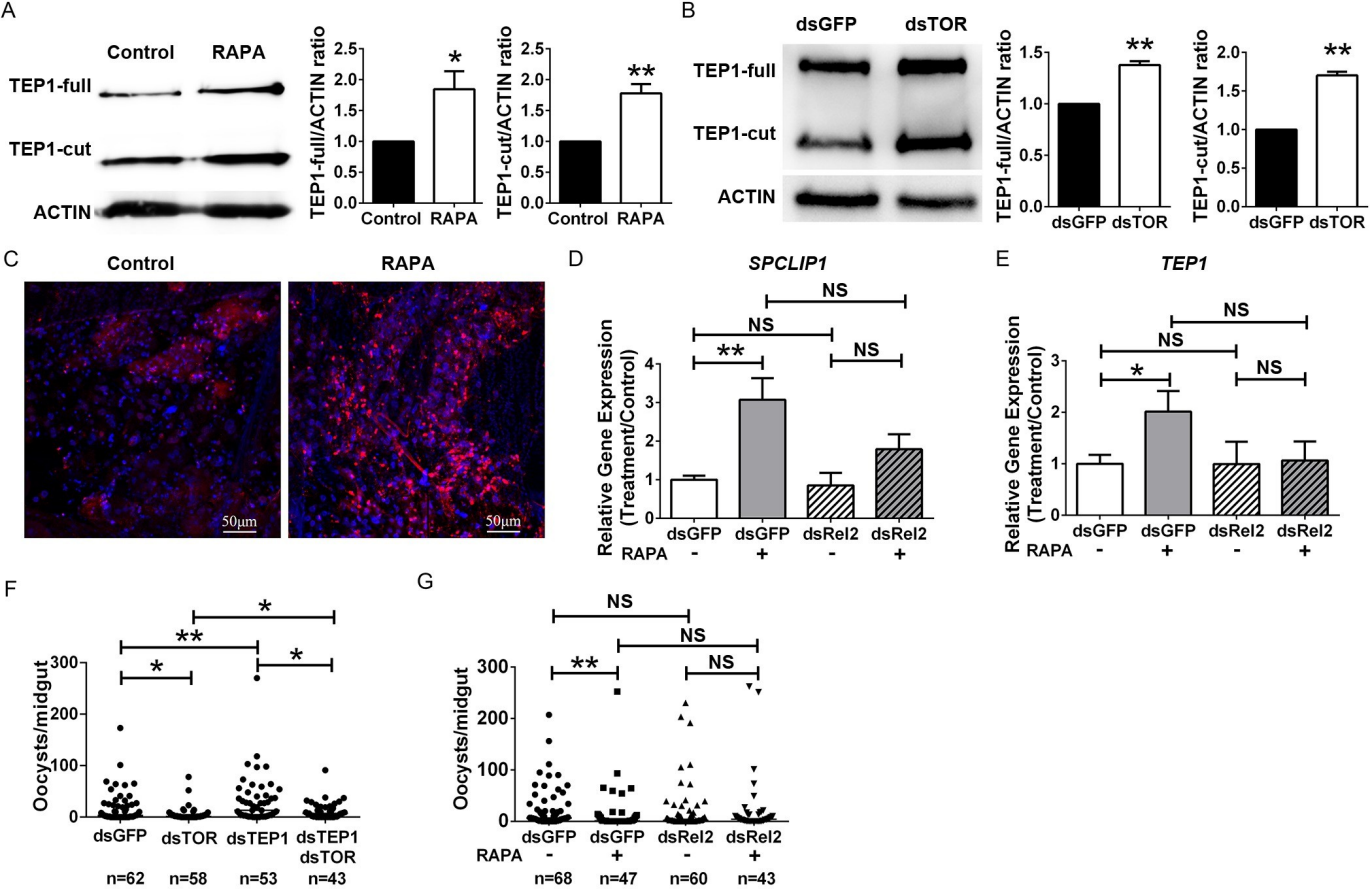
The REL2 is essential in rapamycin mediated increasing resistance to *Plasmodium* infection. Western blot analysis of TEP1 in fat bodies of rapamycin-treated (A) and dsRNA- treated (B) mosquitoes 24 hpi. The blot was probed with the anti-TEP1 polyclonal antibody. *An*. *stephensi* ACTIN was used as the loading control. The bar chart represents relative quantification of signal intensity from at least two independent replicates determined by ImageJ software. Error bars indicate standard errors (n ≥ 2). (C) Whole-mount staining of TEP1 (red) in fat bodies of rapamycin-treated (RAPA) and control (Control) mosquitoes 24 hpi. Nuclei were stained with DAPI (blue). Images are representative of three independent experiments. Scale bars = 50 μm. Influence of REL2 on gene expression of *SPCLIP* (D), and *TEP1* (E) in rapamycin-treated mosquitoes. Expression of target genes was normalized to the reference gene *S7*. Relative gene expression in treated mosquitoes was normalized to that of dsGFP controls. Error bars indicate standard errors (n = 9). Results from one of three independent experiments are shown. (F) Influence of TEP1 on parasite infection. Data were pooled from three independent experiments. (G) Influence of REL2 on vector competence in rapamycin-treated mosquitoes. Data were pooled from at least two independent experiments. Significance was determined by Student’s *t*-test in (A), (B), (D) and (E), and by a Mann-Whitney test in (F, G). *P<0.05, **P<0.01; NS, not significant.

The mosquito NF-κB signaling pathways and complement system are responsible for the majority of *Plasmodium* clearance [27, 28]. TEP1 is the key protein that mediates lysis of malaria parasites by binding to the surface of invading ookinetes [29]. According to our transcriptome results, expression of *TEP1* was significantly induced in rapamycin-treated mosquitoes (Fig 4). We next measured the TEP1 protein level in mosquitoes, of which TOR signaling was inhibited by Western blot and immunohistochemistry analyses. Rapamycin treatment and knockdown of *TOR* both induced protein expression of the full length (TEP1-F) and cleaved form (TEP1-C) of TEP1 by the TEP1-C-terminal-specific antibody (Fig 5A and 5B). Similarly, increased fluorescent signals of TEP1 were observed in the fat bodies of rapamycin-treated mosquitoes (Figs 5C and S8). Given that *TEP1* and *SPCLIP1* were correspondingly induced in most of the rapamycin treatments, and that REL2 controls the transcription of multiple immune effectors, it is possible that upregulation of these immune effectors was due to the enhanced activation of *REL2*. We next specifically knocked down *REL2* and examined the expression levels of *TEP1* and *SPCLIP1* in rapamycin injected mosquitoes 24 hpi (Figs 5D, 5E and S9A). In agreement with our transcriptome results, injection of rapamycin in dsGFP mosquitoes led to a significant increase in the expression of *TEP1* and *SPCLIP1* compared to that of the vehicle-treated controls, while the induction of these two genes was abolished when *REL2* was knocked down (Fig 5D and 5E). However, the expression levels of *SPCLIP1* and *TEP1* were comparable between dsRel2 and dsGFP mosquitoes whether rapamycin was present or not (Fig 5D and 5E). These results indicate that *SPCLIP1* and *TEP1* transcriptional induction by rapamycin injection is mediated by REL2. To examine whether TEP1 induction in the presence of rapamycin could contribute to the increased resistance to *P*. *berghei* infection, we compared the infection outcome among mosquitoes with single knockdown of *TEP1* or *TOR*, and double knockdown of *TEP1* and *TOR*. As expected, the oocyst number was significantly higher in dsTEP1, while lower in dsTOR mosquitoes compared to that in dsGFP ones (Figs 5F and S9B). When both *TOR* and *TEP1* were knocked down, mosquitoes displayed increasing susceptibility to parasite infection compared to dsTOR mosquitoes, but these mosquitoes still had significantly fewer oocysts than dsTEP1 ones did (Fig 5F). These results indicate that other immune effectors in addition to TEP1 controlled by REL2 also contribute to parasite defense in the presence of rapamycin. We next examined the role of REL2 in rapamycin-mediated increased refractoriness to *P*. *berghei* infection. We specifically knocked down *REL2* and analyzed mosquito susceptibility to *P*. *berghei* infection. The infection rate was comparable between dsREL2 and dsGFP mosquitoes either in the presence or absence of rapamycin (Fig 5G). However, knocking down *REL2* completely abrogated the rapamycin-induced resistance to parasite infection that was observed in dsGFP controls (Fig 5G). Altogether, these results indicate that inhibition of the TOR pathway by rapamycin during *Plasmodium* infection induces a dramatic transcriptional reprogramming of the immune defense. The enhanced function of REL2 conferred resistance to *Plasmodium* infection.

## Discussion

Feeding on vertebrate blood initiates vitellogenesis and results in the acquisition and transmission of pathogens in female mosquitoes. The role of the TOR pathway in mosquito vitellogenesis is well defined. However, its influence on vector competence remains unclear. In the current study, we showed that rapamycin introduced into mosquitoes through different approaches, including microinjection, feeding, and surface exposure, all effectively inhibited *Plasmodium* transmission in *Anopheles* mosquitoes. Such an inhibitory effect is realized through boosting the immune response.

Rapamycin was initially isolated from soil samples from Easter Island as an antifungal reagent and then was identified as the inhibitor of the TOR complex in animals [30, 31]. Due to the central role of TOR in health and disease, rapamycin has been used as a drug to treat various human diseases, including allograft rejection, cancer, and neurological diseases [31]. Here we demonstrated that rapamycin effectively inhibited pathogen transmission in mosquitoes through three different approaches. Injection of rapamycin was initially used to examine the role of TOR in vitellogenesis [12]. Our study shows that such an approach also promotes the mosquito’s defense against pathogens. Introduction of rapamycin to mosquitoes through feeding on rapamycin-treated mice was considered because of the protective effect of rapamycin against experimental cerebral malaria in mice [19, 20]. The significant inhibitory effects on the development of oocysts and sporozoites by this approach suggest that it might be a potential adjunctive drug, not only by blocking the development of cerebral malaria but also by preventing the transmission of *Plasmodium* between mammals and *Anopheles* mosquitoes. Similarly, administration of atovaquone, a parasite cytochrome *b* inhibitor, to *P*. *berghei* infected mice prior to *An*. *stephensi* feeding significantly reduced oocysts and sporozoites development in mosquitoes [32]. Here, we also showed that rapamycin effectively increases mortality and reduces fecundity through contact with the tarsi of *An*. *stephensi*. Those mosquitoes died with an undigested blood bolus, and the underlying mechanism is still unknown. It is possible that rapamycin might have blocked the synthesis of hormones or enzymes essential for blood digestion, thereby leading to the mosquitoes’ deaths. The surviving mosquitoes also displayed increasing capacity to eliminate *Plasmodium*. *An*. *gambiae* exposed to an atovaquone-coated surface prevented *P*. *falciparum* development in the midgut, but this does not impact mosquito survival or fecundity [33]. Unlike atovaquone that directly targets *Plasmodium* in mosquitoes, rapamycin treatment suppresses pathogen infection by inhibiting the mosquito TOR pathway that influences both anabolic processes and immune responses.

*Plasmodium* parasite infection elicits profound physiological and behavioral changes in mosquitoes. It induces immune responses, enhances the attraction of mosquitoes to human odor and nectar sources, increases uptake of both sugar and blood, and reduces fecundity [14, 27, 34–38]. Upon infection, mosquitoes may need to reallocate the limited nutrients to balance the tradeoffs between immunity and metabolism to ensure survival. In this study, we demonstrated the antagonistic relationship between the TOR pathway and the immune response. Inhibiting TOR activity induced the expression of the NF-κB transcription factor, REL2, that controls the synthesis of downstream anti-plasmodial immune effectors. Knocking down *REL2* abolished the induction of the immune effectors *TEP1* and *SPCLIP1* by rapamycin, thereby restoring the susceptibility of *An*. *stephensi* to *P*. *berghei* infection. The upregulation of *TEP1* and other anti-plasmodial effectors controlled by REL2 play important roles in defense against *P*. *berghei* when TOR pathway is suppressed [39]. Consistent with our observations, downregulation of TOR signaling by yeast restriction in *Drosophila* boosts innate immune responses and increases resistance to bacterial infection [40]. Inhibition of TOR activity increases ROS levels in *Rhodnius prolixus* [41]. Blocking the TOR pathway may lead to the redistribution of resources towards immune defense, ultimately promoting pathogen clearance. However, we failed to detect changes in phosphorylation level or proteolytic cleavage of REL2 due to the lack of mosquito anti-REL2 antibody. The regulation of REL2 by TOR thus remains unclear. Further research is needed to investigate the impact of TOR signaling on REL2 activity and immune responses.

In addition to being regulated directly by the TOR signaling, the rapamycin- mediated immune activation might be due to the alterations of multiple metabolic pathways. In this study, we also found that rapamycin treatment suppressed the expression of a variety of metabolism-related genes and insulin signaling pathway. Among these, the major yolk protein vitellogenin and lipid transporters *Apolipoprotein II/I* and III are known to promote *Plasmodium* infection via reducing the parasite-killing activity of the immune system [21, 22]. The endogenous ILP4 increases *Plasmodium* infection by inhibiting the expression of anti-parasite genes [25]. The steroid hormone 20-hydroxyecdysone (20E) also participates in the regulation of both mosquito fecundity and vector competence [42]. As a master regulator of the cell’s growth and metabolism, the regulation of mosquito immune activity by TOR is complex. Further dissection of the crosstalk between immunity and metabolism during pathogen infection may provide useful insights for developing novel approaches to vector control. In summary, due to the role of the TOR pathway in controlling both vitellogenesis and pathogen infection in *Anopheles* mosquitoes, inhibition of the TOR pathway may be a potential novel strategy to simultaneously reduce mosquito populations and prevent pathogen transmission.

## Materials and methods

### Ethics statement

All procedures involving *An*. *stephensi* were carried out according to the guidelines for animal care and use of the Fudan University and were permitted by the Animal Care and Use Committee, Fudan University.

### Mosquito maintenance and infection

The *Anopheles stephensi* mosquito (Hor strain) was reared under standard conditions [43]. *Anopheles* infection was completed by allowing mosquitoes to feed on *P*. *berghei* (ANKA)-infected BALB/c mice with 3–5% parasitemia, as described previously [43]. Midguts were dissected and oocysts were counted microscopically eight days post-infection. Salivary glands were dissected and sporozoites were counted 21 days post-infection using a Nikon Eclipse Ni-U microscope at 400× magnification [44].

### Rapamycin treatment

Rapamycin stock solution (20 mM) (Sangon Biotech, Shanghai, China) was prepared in DMSO and diluted to a final working concentration in phosphate-buffered saline (PBS). Four to six-day-old female *An*. *stephensi* were injected with 69 nl of 20 μM rapamycin intrathoracically. Age-matched vehicle control solution-injected mosquitoes were used as controls. Infectious blood was offered 12 h post-injection. Treatment of mice with rapamycin was conducted as previously described [19, 45]. Briefly, the stock solution of rapamycin was prepared in ethanol (25 mg/ml). For intravenous injection, the stock solution was diluted to a final concentration of 0.1 mg/ml in a solution of 5% polyethylene glycol 400, 4% ethanol, and 5% Tween 80. *P*. *berghei* infected mice with 3–5% parasitemia were injected with rapamycin via the tail vein at 1 mg/kg, and mosquitoes were allowed to feed 15 min post-rapamycin administration. Exposure to rapamycin-treated Petri dishes was performed as described previously with slight modification [33]. The 6-cm diameter Petri dishes were coated with 2 ml ethanol containing 0.2 mg, 2 mg, and 10 mg rapamycin with the final concentrations 0.077, 0.77, and 3.85 mmol/m^2^, respectively. An equal volume of ethanol was coated on Petri dishes as a control. The dishes were allowed to dry at room temperature and then were placed on the top of a paper cup containing mosquitoes for 6–60 min. Dishes were removed after exposure. Mice infected with *P*. *berghei* were supplied for mosquito feeding. For administration of rapamycin through water feeding, rapamycin stock solution prepared in DMSO was diluted to 20 μM in deionized water. Mosquitoes fed ad libitum on rapamycin-containing water for 12 h, followed by feeding on *P*. *berghei* infected mice. An equal volume of DMSO diluted in deionized water was used as a control.

### Mosquito fecundity and survival

The fecundity analysis was performed by counting the number of mature eggs in ovaries 48 h post-blood meal and eggs deposited on the filter paper three days post-blood meal under a stereo microscope [46]. Survival was checked daily after rapamycin treatment. When a dead mosquito was observed, it was removed from the paper cup.

### RNA interference

The dsRNA products were prepared as previously described [43]. The cDNA clones *TOR*, *REL2*, *TEP1* and plasmid eGFP (BD Biosciences) served as templates for amplification using gene-specific primers (S2 Table). Four to six-day-old females were injected intrathoracically with 69 nl of 3.5 μg/μl dsREL2, dsTEP1, 5.8 μg/μl dsTOR or the mixture of dsTEP1 and dsTOR using a Nanoject II microinjector (Drummond). Equal amounts of dsGFP were injected as a control. Silencing efficiency was examined two days post-dsRNA treatment by quantitative PCR as described below.

### Generation of polyclonal antibodies

The anti-S6 kinase (S6K) rabbit polyclonal antibody was prepared against recombinant S6K corresponding to bases 49–1680 of *s6k* CDS (ASTEI01297) expressed in pET-28a (Novagen). Purified recombinant protein was used to generate the antibodies commercially (GL Biochem Ltd, Shanghai, China). The anti-TEP1 rabbit polyclonal antibody was prepared as previously described [47]. The recombinant TEP1 of *An*. *stephensi* corresponding to bases 3280–3963 of *TEP1* CDS (ASTE016444) was expressed in pET-42a (Novagen). Purified recombinant protein was used to generate antibodies (GL Biochem Ltd, Shanghai, China).

### Transcriptome analysis

*An*. *stephensi* treated with rapamycin or vehicle control solution were collected 24 h post-infection. Midguts were removed to eliminate mammalian blood contamination. Ten of the carcasses were pooled for one biological replicate. RNA of three biological replicates of each treatment was prepared. RNA samples were sent to Novogene, China, for further sequencing and data analysis. Briefly, after removing reads containing adapter, ploy-N, and low-quality reads, clean data were aligned to the *An*. *stephensi* reference genome (https://www.vectorbase.org/organisms/anopheles-stephensi) using Hisat2 v2.0.4 [48]. Differential expression analysis between sample groups was performed using the EdgeR package [49]. Genes with adjusted P-value < 0.05 were considered significantly differentially expressed. Gene Ontology (GO) enrichment analysis of differentially expressed genes was implemented by the GOseq R package in which gene length bias was corrected [50].

### Western blot

Fat bodies of mosquitoes were dissected 12 h or 24 h post blood meal. Proteins of 10 mosquito fat bodies were extracted in 100 μl lysis buffer (50 mM Tris, pH 7.4; 1% IGEPAL 0.25% sodium deoxycholate; 150 mM NaCl; 1 mM EDTA; 1 mM phenylmethylsulfonyl fluoride; 1× protease inhibitor mixture; 1× phosphatase inhibitor mixture) [9]. Immunoblotting was performed using standard procedures. Antibodies used for TOR signaling were rabbit anti-phospho-S6K (Thr398) (1:1000) (Cell Signaling), rabbit anti-S6K (1:1000), and rabbit anti-actin (1:2000) (Sungenebiotech, China). Protein used for immunoblotting for p-Akt was extracted from fat bodies/ovaries, and midgut 12 hpi. The p-Akt was detected using a Phospho-Akt (Ser473) Antibody (1:200) (Cell Signaling) [51]. Immunoblotting for TEP1 was performed similarly, except that proteins from ten whole mosquitoes were extracted in cracking buffer (8 M urea, 2% SDS, 5% β-mercaptoethanol, 125 mM Tris-HCl) and 1:1000 anti-TEP1 rabbit polyclonal antibody was used. Intensity of the signals was quantified by ImageJ software [52].

### Immunohistochemistry

The fat bodies of *An*. *stephensi* 24 hpi were fixed in 4% paraformaldehyde for 2 h at 4°C, followed by three 10-min washes in PBS containing 0.1% Trixon-100. After blocking in 3% BSA for 2 h at 4°C, the tissues were incubated with anti-TEP1 rabbit polyclonal antibody (1:1000 dilution) or pre-immune sera overnight at 4°C. The secondary antibody, Alexa Fluor 546 (Invitrogen), was used at 1:1000 dilution. The nucleus was stained with 10 μg/μl DAPI (Solarbio, China). Images were acquired by a Zeiss LSM710 confocal microscope connected to a Nikon Digital Sight DS-U3 digital camera.

### Quantitative PCR

For gene expression analysis in *An*. *stephensi*, total RNA was extracted from mosquitoes 24 hpi utilizing the TRIzol method (Sigma-Aldrich, China). Reverse transcription and quantitative PCR were performed as previously described [43]. The expression levels of target genes were normalized by the *An*. *stephensi* ribosomal gene *S7*. For detection of the abundance of gut microbiota, three midguts were pooled for DNA extraction. The levels of *16S rRNA* gene were determined by quantitative PCR. The primers used for this study are listed in S2 Table.

### Statistical analysis

Replicates and sample sizes for all experiments are provided in the corresponding figure legends. All statistical analyses were performed using GraphPad Prism software (v.8). Averages from data with non-normal distributions are shown as medians, and averages from data with normal distributions are shown as means with standard errors. The Mann-Whitney test was used to compare non-normally distributed data, and Student’s *t*-test was used to compare normally distributed data. A Log-rank (Mantel-Cox) test was performed to compare the survival curves of *An*. *stephensi* exposed to rapamycin and control solution. All source data were shown in S3 Table.

## Supporting information

S1 Fig *TOR* silencing efficiency in dsRNA-treated *An*. *stephensi*. The expression level of *TOR* was normalized to *S7*. The relative expression level of *TOR* in dsTOR mosquitoes was normalized to the gene’s expression in dsGFP controls. Error bars indicate standard errors (n = 6). Results from one of three independent experiments are shown.
(TIF)

S2 Fig Parasitemia of mice treated with rapamycin or vehicle control solution. Error bars indicate standard errors. Results from one of three independent experiments are shown.
(TIF)

S3 Fig Oocyst numbers of mosquitoes exposed to a rapamycin-coated surface. Oocyst numbers of mosquitoes exposed to 3.85 mmol/m^2^ (A) and 0.077 mmol/m^2^ (B) rapamycin (red dots) or solvent (black dots) coated surfaces for 60 min. Data were pooled from two independent experiments. Horizontal black bars indicate the median values.
(TIF)

S4 Fig Oocyst numbers of mosquitoes orally supplemented with rapamycin-containing water. Data were pooled from two independent experiments. Horizontal black bars indicate the median values.
(TIF)

S5 Fig GO enrichment analysis of differentially expressed genes. BP, biological process; CC, cellular component; MF, molecular function.
(TIF)

S6 Fig Rapamycin microinjection inhibits nutrient transportation and insulin signaling. (A) Relative gene expression of *Vitellogenin (Vg)*, *Apolipoprotein II/I* (*Apo II/I*), and *Apolipoprotein III (Apo III)* in the fat bodies of rapamycin-injected *An*. *stephensi* at 24 hpi (n = 10). Western blot analysis of Akt phosphorylation in fat bodies/ovaries (B), and midguts (C) collected from mosquitoes at 12 hpi. Results from one of two independent experiments are shown. **P<0.01, ****P< 0.0001.
(TIF)

S7 Fig Abundance of microbiota in rapamycin-injected mosquitoes. Quantification of *16S rRNA* gene in the midgut of rapamycin-injected *An*. *stephensi* prior to a blood meal (A) or at 24 hpi (B). The *16S rRNA* gene level was normalized to *S7*. Error bars indicate standard errors (n = 10). Results from one of two independent experiments are shown. Significance was determined by Student’s *t*-test.
(TIF)

S8 Fig Whole-mount staining of TEP1 with pre-immune serum. Fat bodies of rapamycin-treated (RAPA) and control (Control) mosquitoes 24 hpi were stained with pre-immune serum. Nuclei were stained with DAPI (blue). Images are representative of three independent experiments. Scale bars = 50 μm.
(TIF)

S9 Fig The knockdown efficiency of *REL2* and *TEP1*. (A) Relative expression levels of *REL1* and *REL2* in dsRel2 mosquitoes were normalized to those in dsGFP controls. Error bars indicate standard errors (n = 8). (B) Relative expression levels of *TEP1* in dsGFP and dsTEP1 were normalized to *S7*. The relative gene expression level in treated mosquitoes was normalized to the gene’s expression in dsGFP controls. Error bars indicate standard errors (n = 8). Results from one of threat least two independent experiments are shown. Significance was determined by Student’s-*t* test; **P<0.01.
(TIF)

S1 Table List of differentially regulated genes. (XLSX)

S2 Table Primers used for PCR amplification. (DOCX)

S3 Table Source Data. (XLSX)
