# Supplementary figures and images for: Rapamycin inhibits pathogen transmission in mosquitoes by promoting immune activation
Source: PLoS Pathog. 2021 Feb 24;17(2):e1009353. doi: 10.1371/journal.ppat.1009353 (PMC7939355; doi:10.1371/journal.ppat.1009353)

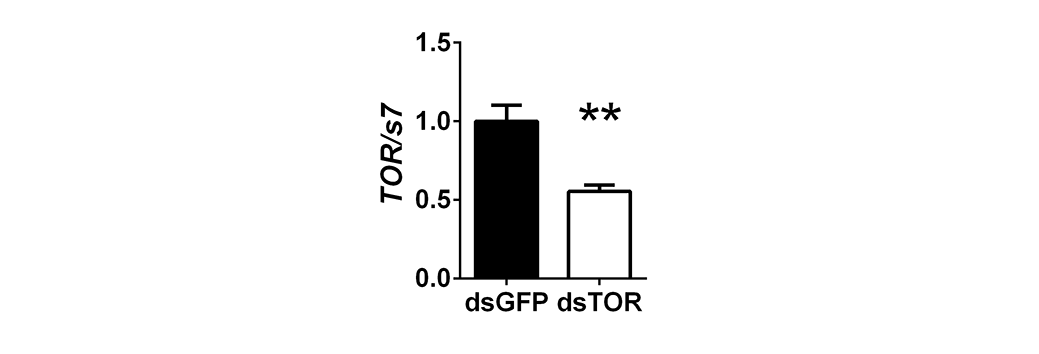

Supplement: S1 Fig — The expression level of TOR was normalized to S7. The relative expression level of TOR in dsTOR mosquitoes was normalized to the gene’s expression in dsGFP controls. Error bars indicate standard errors (n = 6). Results from one of three independent experiments are shown. (TIF) [file ppat.1009353.s001.tif]

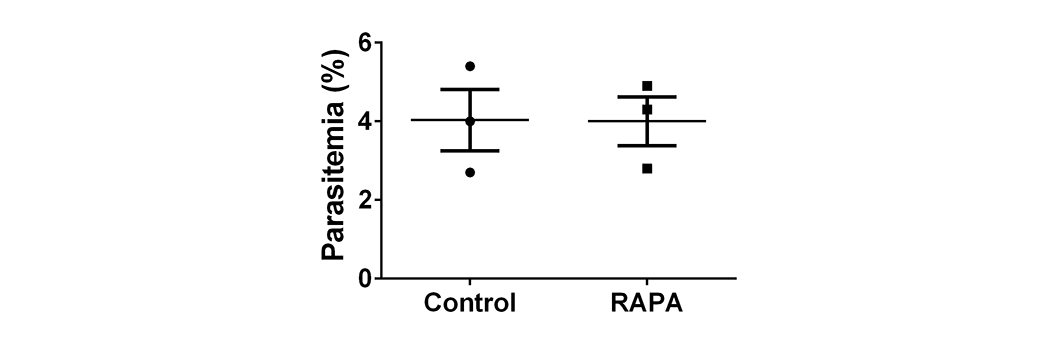

Supplement: S2 Fig — Error bars indicate standard errors. Results from one of three independent experiments are shown. (TIF) [file ppat.1009353.s002.tif]

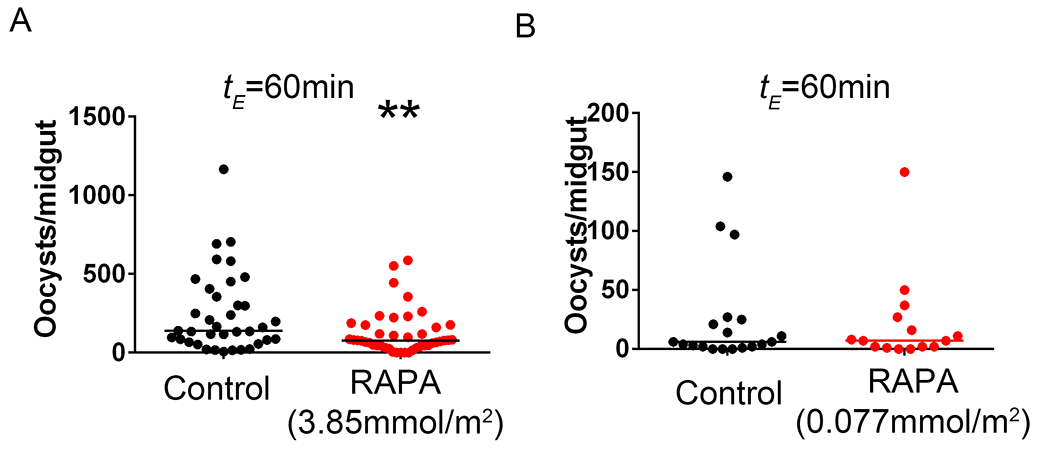

Supplement: S3 Fig — Oocyst numbers of mosquitoes exposed to 3.85 mmol/m2 (A) and 0.077 mmol/m2 (B) rapamycin (red dots) or solvent (black dots) coated surfaces for 60 min. Data were pooled from two independent experiments. Horizontal black bars indicate the median values. (TIF) [file ppat.1009353.s003.tif]

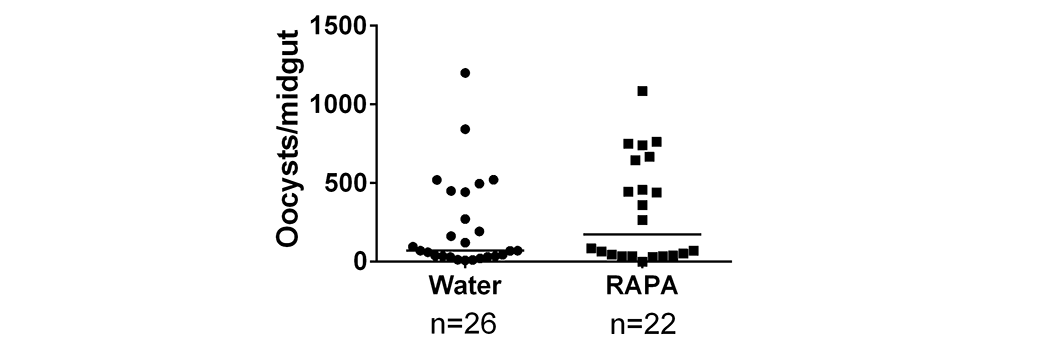

Supplement: S4 Fig — Data were pooled from two independent experiments. Horizontal black bars indicate the median values. (TIF) [file ppat.1009353.s004.tif]

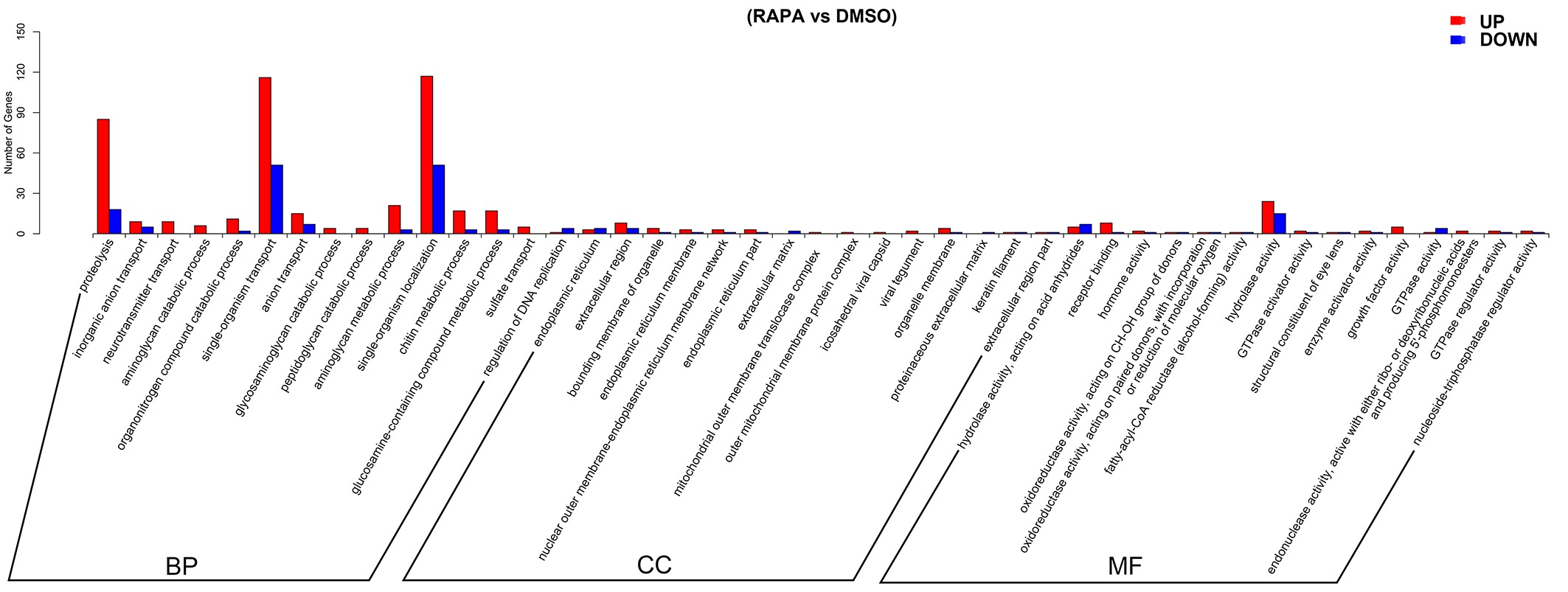

Supplement: S5 Fig — BP, biological process; CC, cellular component; MF, molecular function. (TIF) [file ppat.1009353.s005.tif]

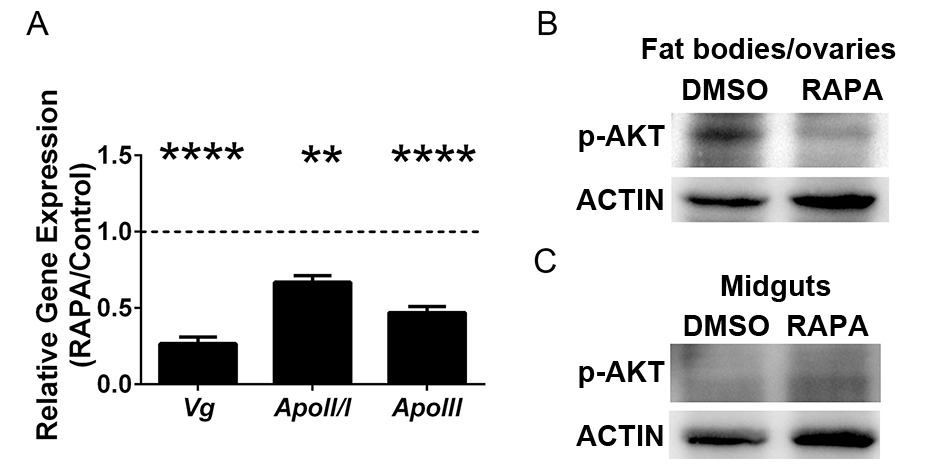

Supplement: S6 Fig — (A) Relative gene expression of Vitellogenin (Vg), Apolipoprotein II/I (Apo II/I), and Apolipoprotein III (Apo III) in the fat bodies of rapamycin-injected An. stephensi at 24 hpi (n = 10). Western blot analysis of Akt phosphorylation in fat bodies/ovaries (B), and midguts (C) collected from mosquitoes at 12 hpi. Results from one of two independent experiments are shown. **P<0.01, ****P< 0.0001. (TIF) [file ppat.1009353.s006.tif]

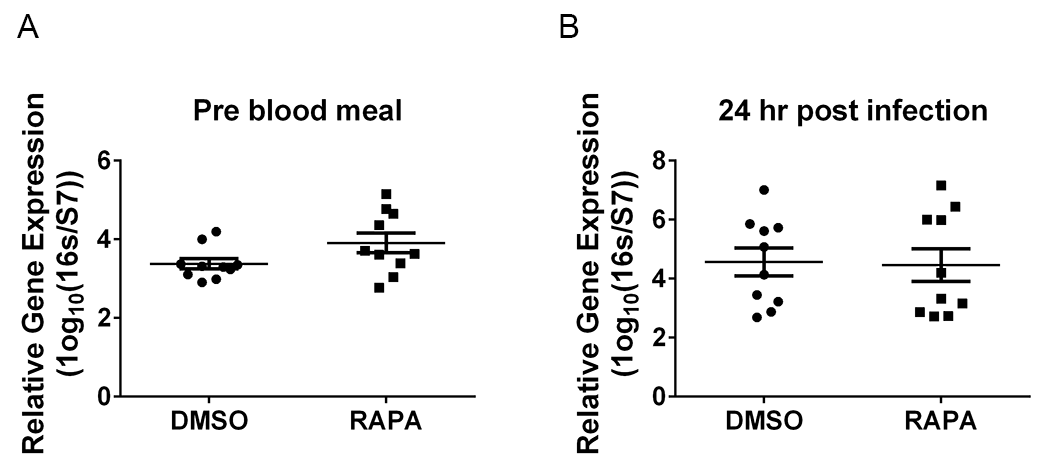

Supplement: S7 Fig — Quantification of 16S rRNA gene in the midgut of rapamycin-injected An. stephensi prior to a blood meal (A) or at 24 hpi (B). The 16S rRNA gene level was normalized to S7. Error bars indicate standard errors (n = 10). Results from one of two independent experiments are shown. Significance was determined by Student’s t-test. (TIF) [file ppat.1009353.s007.tif]

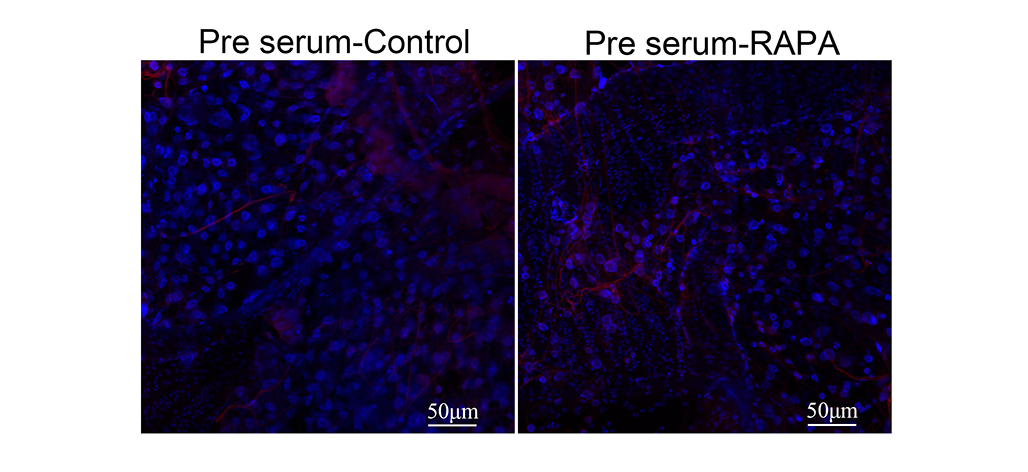

Supplement: S8 Fig — Fat bodies of rapamycin-treated (RAPA) and control (Control) mosquitoes 24 hpi were stained with pre-immune serum. Nuclei were stained with DAPI (blue). Images are representative of three independent experiments. Scale bars = 50 μm. (TIF) [file ppat.1009353.s008.tif]

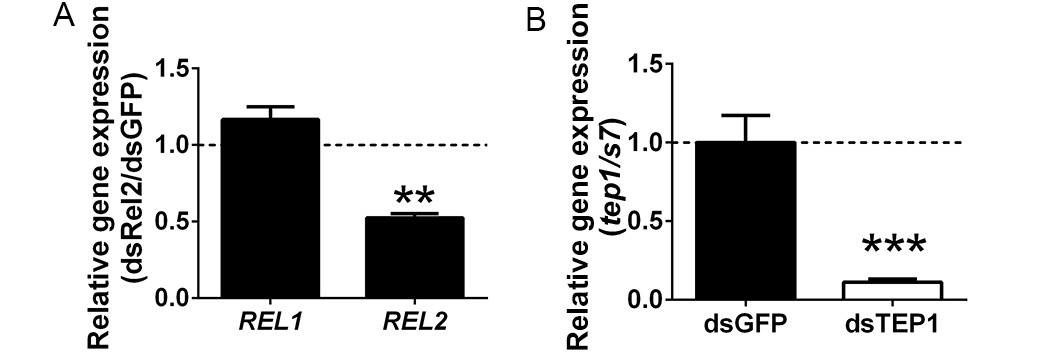

Supplement: S9 Fig — (A) Relative expression levels of REL1 and REL2 in dsRel2 mosquitoes were normalized to those in dsGFP controls. Error bars indicate standard errors (n = 8). (B) Relative expression levels of TEP1 in dsGFP and dsTEP1 were normalized to S7. The relative gene expression level in treated mosquitoes was normalized to the gene’s expression in dsGFP controls. Error bars indicate standard errors (n = 8). Results from one of threat least two independent experiments are shown. Significance was determined by Student’s-t test; **P<0.01. (TIF) [file ppat.1009353.s009.tif]
