# Supplementary material for: Rapamycin inhibits pathogen transmission in mosquitoes by promoting immune activation
Source: PLoS Pathog. 2021 Feb 24;17(2):e1009353. doi: 10.1371/journal.ppat.1009353 (PMC7939355; doi:10.1371/journal.ppat.1009353)
Supplement: S2 Table — (DOCX) [file ppat.1009353.s011.docx]

**S2 Table.** **Primers used for PCR amplification.**

| Primer  (Accession#) | Primer sequences (F, Forward; R, reverse) | Amplicon (bp) |
| --- | --- | --- |
| qS7  (ASTE004816) | F: 5' TGCGGAGCGTCGTATTCTGC 3'  R: 5' ACACAGCGGTGAGCGTTCG 3' | 79 |
| T7-GFP^∆^  (BD Biosciences) | F: 5' TAATACGACTCACTATAGGGTCAGTGGAGAGGGTGAAG 3'  R: 5' TAATACGACTCACTATAGGCTAGTTGAACGGATCCATC 3' | 454 |
| T7-dsTOR^∆^  (ASTE011722) | F: 5' TAATACGACTCACTATAGGGCGGTGCTGGAAGCGTTTGT 3'  R: 5' TAATACGACTCACTATAGGTTACCAAAATGGGCACCACCCGATG 3' | 584 |
| \| qTOR \| \| --- \| | F: 5' CAGACAGAGGAGCACCACCAGT 3'  R: 5' TCGTACCATCGCACCTGAACCT 3' | 152 |
| qTEP1  (ASTE016444) | F: 5' GCCTTGCTGTCGTTCGTGAT 3'  R: 5' CCTGGGTGCGTGGGAAAC 3' | 106 |
| T7-dsTEP1^∆^ | F: TAATACGACTCACTATAGGTCGGGCTGAAGGCGTTGA  R: TAATACGACTCACTATAGG TGCCACCTTGAATCGTCTGA | 583 |
| TEP1* | F: 5' GGGCTGAAGGCGTTGA 3'  R: 5' TTATGCTTTTGCGCACTCC 3' | 684 |
| EcoRI-TEP1*  Xhol-TEP1* | F: 5' TCCGAATTCGGGCTGAAGGCGTTGA 3'  R: 5' GTGCTCGAGTTATGCTTTTGCGCACTCC 3' | 702 |
| qLA  (ASTE002619) | F: 5' ACGCAGCCATCGGTGAGC 3'  R: 5' GCAGACGGACAGTGTTCGGTTT 3' | 131 |
| qLB  (ASTE006009) | F: 5' GGCGATTGGGTTGCGGATTT 3'  R: 5' CGATGTCCGAGCAGGGTGTA 3' | 116 |
| qAPL1  (ASTE016290) | F: 5' AGAGTCGGCAGGCGTTCAA 3'  R: 5' GCTTGTCGGTCTTCAGGGTCAG 3' | 135 |
| qSPCLIP1  (ASTE008194) | F: 5' GGTTGCTTTCTGCGGATGATTTCG 3'  R: 5' CGGCTCTTCGGTGGTTGTCA 3' | 128 |
| qREL2  (ASTE010360) | F: 5' AACAGCAGCAACAGCATTACT 3'  R: 5' TGATAGCGGAAGCGGAACT 3' | 184 |
| T7-dsREL2^∆^ | F: 5' TAATACGACTCACTATAGGAACGGCCGGTGACGATAGT 3'  R: 5' TAATACGACTCACTATAGGCTACTTCATCCGTCGACAG 3' | 600 |
| qCecropin a  (ASTE007107) | F: 5' GCGGTACTGCTGCTCTGC 3'  R: 5' GCTTGCCAACTCCTTCAATCTTCT 3' | 79 |
| qAttacin  (ASTE009529) | F: 5' CGCCTCACCATTGTCAAGCCAAAT 3'  R: 5' CGTCCGTTCCGTATCCGTCCTC 3' | 126 |
| qVg  (ASTE003745) | F: 5' AGTCGTCCTCGTCGTCGTCTT 3'  R: 5' GCGGTTCGGCTTCTGTTCCT 3' | 89 |
| qApoII/I  (ASTE006592) | F: 5' AGATGCGACCGTTGTGGAGAAGT 3'  R: 5' TTGATGCCTGCCTTGCTGTTGAC 3' | 108 |
| qApoIII  (ASTE001437) | F: 5' GGTGAAGAAGCATTCGGACAT 3'  R: 5' CAGGGTGGCAGTGGTTTG 3' | 79 |
| q16S rDNA | F: 5' TGGAGCATGTGGTTTAATTCGA 3'  F: 5' TGCGGGACTTAACCCAACA 3' | 143 |
| S6k*  (ASTEI01297) | F: 5' GTCGACCTCGAACCCGAACTT 3'  R: 5' AGAGATGGACATTTCCGGATACACA 3' | 1632 |
| BamHI-S6k*  Xhol-S6k* | F: 5' CGCGGATCCGTCGACCTCGAACCCGAACTT 3'  R: 5' GTGCTCGAGAGAGATGGACATTTCCGGATACACA 3' | 1650 |

*: Primers for polyclonal antibody generation

^∆^: Primers for dsRNA synthesis

q: Primers for real time quantitative PCR
